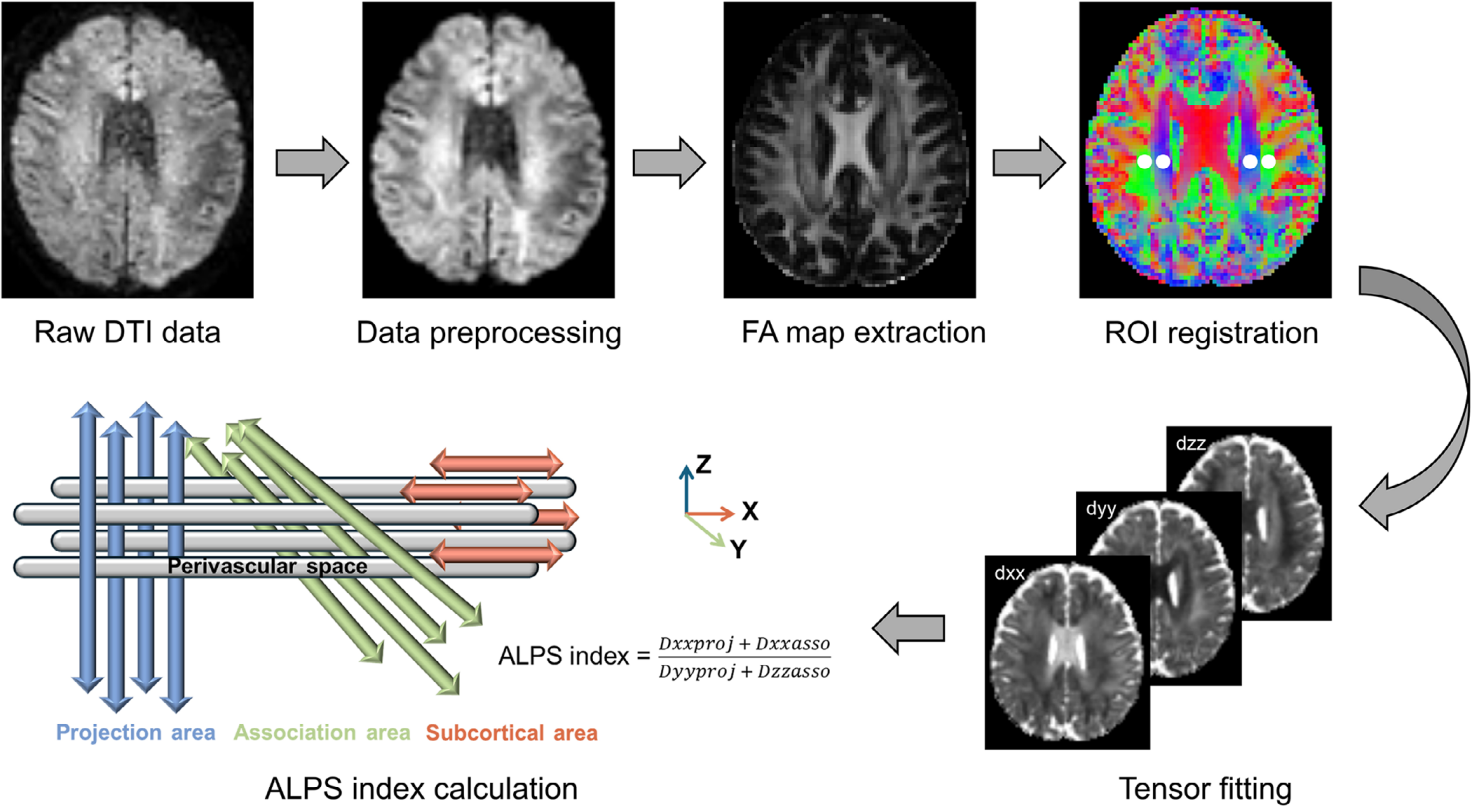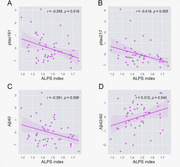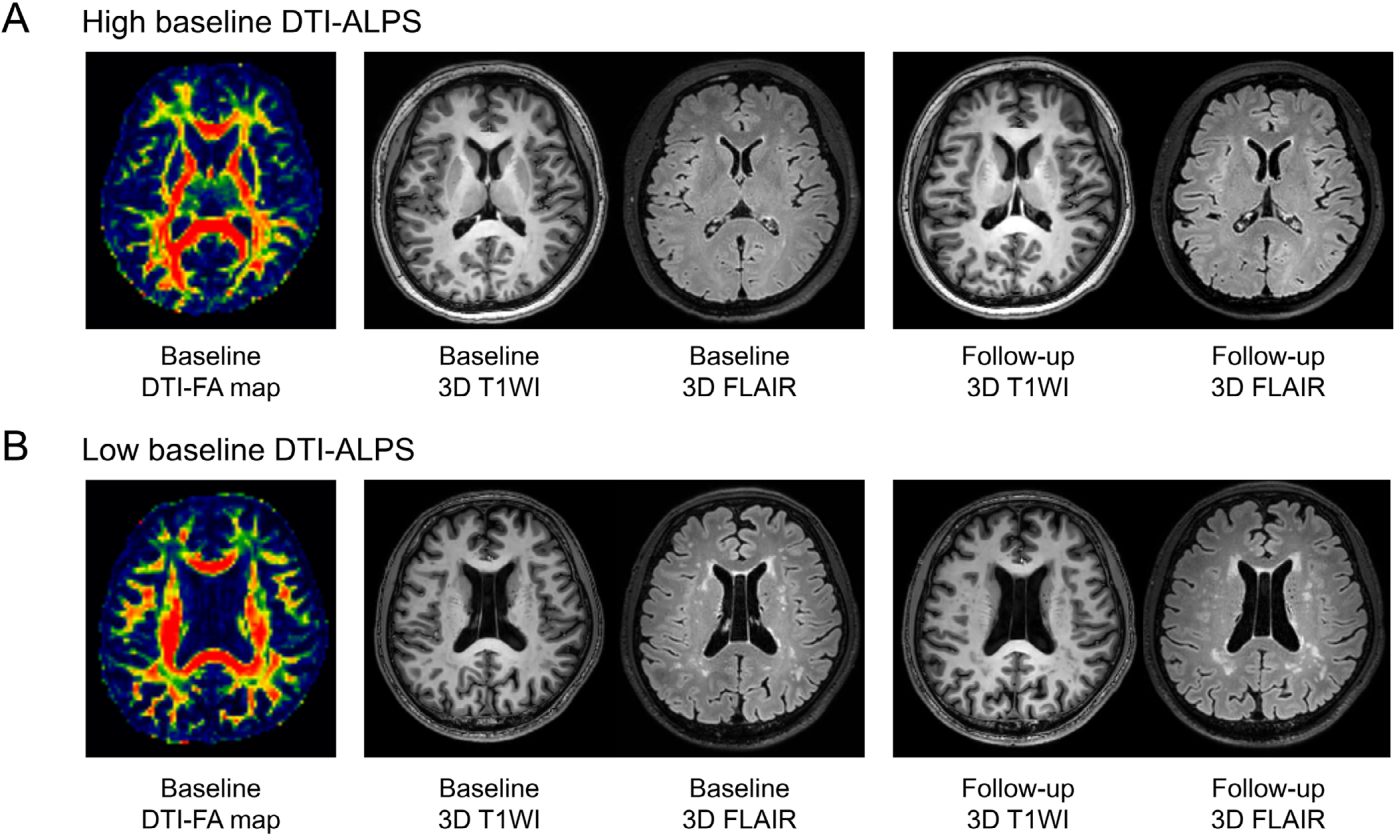# Glymphatic system function associates with AD‐signature region volumes, plasma biomarkers, and white matter hyperintensity progression in cognitively unimpaired elderly

**DOI:** 10.1002/alz70856_097011

**Published:** 2025-12-24

**Authors:** Qian Chen, Bing Zhang

**Affiliations:** ^1^ Department of Radiology, Nanjing Drum Tower Hospital, Affiliated Hospital of Medical School, Nanjing University, Nanjing, Jiangsu, China; ^2^ Nanjing Drum Tower Hospital, Nanjing, Jiangsu, China

## Abstract

**Background:**

The glymphatic system, the brain's waste clearance mechanism, is believed to play a critical role in the pathogenesis of Alzheimer's disease (AD). Glymphatic dysfunction may lead to β‐amyloid accumulation, followed by tau phosphorylation and neuronal degeneration, ultimately contributing to cognitive decline and dementia. Diffusion tensor imaging along the perivascular space (DTI‐ALPS) provides a noninvasive and effective means to assess glymphatic system activity. However, the relationships between the DTI‐ALPS index, volumes of AD‐signature regions, plasma biomarkers, and disease progression in cognitively unimpaired elderly individuals remain poorly understood.

**Method:**

A total of 229 cognitively unimpaired elderly individuals were enrolled across two datasets. The associations between the DTI‐ALPS index and volumes of AD‐signature regions, including the basal forebrain, entorhinal cortex, and hippocampus, as well as white matter hyperintensity (WMH) volumes, were evaluated. In dataset 1 with plasma biomarkers, the mediating effects of the DTI‐ALPS index on the relationship between plasma biomarkers and cognition were assessed. In dataset 2 with follow‐up data (1.5 years ± 12 months), the baseline DTI‐ALPS index was correlated with the annual percent change (APC) in both AD‐signature region and WMH volumes.

**Result:**

The DTI‐ALPS index exhibited positive correlations with volumes in the basal forebrain, entorhinal cortex, and hippocampus, and negative correlations with white matter hyperintensity (WMH) volumes in both datasets. It was negatively associated with plasma ptau181, ptau217, and Aβ40, while positively associated with the Aβ42/40 ratio. The DTI‐ALPS index mediated the relationship between plasma ptau181 and MMSE scores, as well as between ptau217 and MMSE scores. Follow‐up data revealed no significant correlations between the baseline DTI‐ALPS index and the APC in the volumes of the basal forebrain, entorhinal cortex, and hippocampus. However, baseline DTI‐ALPS was negatively correlated with the APC of WMH (*r* = ‐0.387, *p* = 0.006).

**Conclusion:**

Impaired glymphatic system function is associated with reduced volumes in AD‐signature regions, more severe WMH lesions, higher plasma ptau levels, and accelerated WMH progression, before the onset of objective cognitive impairment. Therapeutic approaches targeting the glymphatic system could potentially prevent cognitive decline by promoting the clearance of AD‐related pathological proteins and slowing the progression of WMH lesions.